# Mortality in Women across the *FMR1* CGG Repeat Range: The Neuroprotective Effect of Higher Education

**DOI:** 10.3390/cells12172137

**Published:** 2023-08-24

**Authors:** Jinkuk Hong, Robert S. Dembo, Leann Smith DaWalt, Mei Wang Baker, Elizabeth Berry-Kravis, Marsha R. Mailick

**Affiliations:** 1 Waisman Center, University of Wisconsin-Madison, Madison, WI 53705, USA; rdembo@wisc.edu (R.S.D.); lesmith2@wisc.edu (L.S.D.); marsha.mailick@wisc.edu (M.R.M.); 2 NORC at the University of Chicago, Chicago, IL 60603, USA; 3 Department of Pediatrics, School of Medicine and Public Health, University of Wisconsin-Madison, Madison, WI 53792, USA; mei.baker@slh.wisc.edu; 4 Wisconsin State Laboratory of Hygiene, Madison, WI 53706, USA; 5 Department of Pediatrics, Rush University Medical Center, Chicago, IL 60612, USA; elizabeth_berry-kravis@rush.edu; 6 Department of Neurological Sciences, Rush University Medical Center, Chicago, IL 60612, USA; 7 Department of Anatomy and Cell Biology, Rush University Medical Center, Chicago, IL 60612, USA

**Keywords:** *FMR1* CGG repeats, mortality, higher education, differential sensitivity

## Abstract

Higher education has been shown to have neuroprotective effects, reducing the risk of Alzheimer’s and Parkinson’s diseases, slowing the rate of age-related cognitive decline, and is associated with lower rates of early mortality. In the present study, the association between higher education, fragile X messenger ribonucleoprotein 1 (*FMR1*) cytosine–guanine–guanine (CGG) repeat number, and mortality before life expectancy was investigated in a population cohort of women born in 1939. The findings revealed a significant interaction between years of higher education and CGG repeat number. Counter to the study’s hypothesis, the effects of higher education became more pronounced as the number of CGG repeats increased. There was no effect of years of higher education on early mortality for women who had 25 repeats, while each year of higher education decreased the hazard of early mortality by 8% for women who had 30 repeats. For women with 41 repeats, the hazard was decreased by 14% for each additional year of higher education. The interaction remained significant after controlling for IQ and family socioeconomic status (SES) measured during high school, as well as factors measured during adulthood (family, psychosocial, health, and financial factors). The results are interpreted in the context of differential sensitivity to the environment, a conceptualization that posits that some people are more reactive to both negative and positive environmental conditions. Expansions in CGG repeats have been shown in previous *FMR1* research to manifest such a differential sensitivity pattern.

## 1. Introduction

Research on genotype–phenotype associations in *FMR1* has focused primarily on those with fragile X syndrome (FXS), the premutation, and to a lesser extent the gray zone. Almost all of this research has aimed to reveal how those at the higher ends of the repeat distribution differ from the norm, and the focus has been on pathological functioning, including fragile X-associated tremor/ataxia syndrome (FXTAS), fragile X-associated premature ovarian insufficiency (FXPOI), and fragile X-associated neuropsychiatric disorders (FXAND) [1,2].

Most genotype–phenotype associations in *FMR1* have been based on data from clinical populations, collected following the diagnosis of a child with FXS when the family may be tested to determine if each member has expansions in the number of CGG repeats. Thus, the full variation in CGG repeat number is not evaluated in these studies because only those at the higher end of the repeat distribution, and their relatives, are generally assayed. However, the number of CGG repeats in *FMR1* is highly polymorphic in the human population [3,4]. The population mode for CGG repeats is at 30, with >90% of individuals having fewer than 40 and the lowest reported being 6 repeats [4,5,6,7]. Thus, understanding the genotype–phenotype associations of *FMR1* polymorphisms in the vast majority of the population is of great interest in order to fill the knowledge gap.

Expansions of CGG repeats have been associated with health and cognitive limitations. Here we define expansions starting at 41 repeats, which many studies have defined as the lower limit of the gray zone [8,9,10,11], and our analysis extends through the low premutation range. Premutation carriers are at risk of neurocognitive, motor, psychiatric, and reproductive symptoms, for which there are no specific pharmaceutical treatments, although symptomatic treatments can be effective. Less is known about the prevalence of symptoms in individuals in the gray zone, although there is a growing literature indicating the risk of symptoms [12,13]. The present study aims to determine whether non-clinical approaches can reduce the health risks that have been associated with CGG expansions in a population-based sample. Here we focus on the protective effects of post-secondary education, which have been associated with a range of beneficial health outcomes in the general population, including longevity [14,15]. In this study, we ask whether post-secondary education is associated with reduced rates of early mortality (i.e., mortality before life expectancy) in women who have between 7 and 84 CGG repeats. We further ask whether education effects differ by CGG repeat number.

### 1.1. Effects of Higher Education on Mortality

Several mechanisms have been proposed to explain the educational gradient in mortality in the general population, including income, psychosocial resources, and health behaviors [16]. Some have argued that cognitive functioning is the most important pathway through which education improves health and reduces mortality risk [17]. Education provides the context for cognitively stimulating activities, and those with higher levels of education tend to exhibit greater cognitive functioning throughout adulthood than those with lower levels of education (for a review, see [18]).

Studies have shown an association between educational attainment and reduced or delayed symptoms of Alzheimer’s disease and dementia [19,20], Parkinson’s disease [21], as well as other neurological signs and symptoms [22], and verbal and working memory [23]. Taken together, these findings suggest that post-secondary education contributes to cognitive reserve and may have broader neuroprotective effects.

A growing area of research explores how educational attainment might interact with genetic factors to predict differential patterns of health. For example, there is evidence to suggest that post-secondary education may help offset the genetic predisposition to certain health problems, including type 2 diabetes [24], abnormally high body mass index [25], impaired kidney function [26], and cognitive decline [27]. These findings reflect a pattern of “differential sensitivity to the environment”, wherein individuals with certain genetic profiles have a heightened sensitivity to environmental exposures (e.g., post-secondary education [28,29]).

With respect to the *FMR1* gene, only a few prior studies have reported data on the beneficial effects of higher education with respect to health. In these studies, findings indicate that those with higher levels of education have better motor, cognitive [30], and executive functioning [31], a lower prevalence of FXTAS [32,33], and fewer related neurological symptoms [34]. All of these studies were based on clinically-ascertained samples, with no prior studies examining whether the benefits of higher education vary by CGG repeat length in a general population sample.

This investigation builds on prior research regarding the effect of higher education in reducing the genetic predisposition to certain health problems. In this study, we examine whether the mortality effect of post-secondary education varies according to the *FMR1* CGG repeat number. A key question is the extent to which this effect is due to neuroprotection and enhanced cognitive reserve or to the cumulative advantages and disadvantages that occur after the completion of education. If the effect of higher education is primarily neuroprotective, then the independent effect of the interaction between higher education and CGG repeats in predicting mortality should persist even when indicators of cumulative advantage/disadvantage are controlled. For the present study, we focused on four domains that have been proposed to explain the educational gradient in mortality in the general population: family factors, psychosocial factors, health, and financial factors.

### 1.2. Family Factors

Marriage. The relationship between marital status and mortality is one of the most studied empirical associations, with research consistently finding a protective effect of marriage on a range of health outcomes [35] as well as all-cause mortality [36]. In one meta-analysis, unmarried individuals were found to have a 24% higher risk of early mortality than those who were married [37].

Parental death. Prior research has found a strong association between parental death and a range of health outcomes in surviving children both during childhood and adulthood [38,39]. Further, there is evidence to suggest that an individual’s own mortality is influenced by parental longevity, partly as a result of shared genetic vulnerability [40]. The death of both parents may elevate this risk to a greater degree [41,42], even in midlife.

### 1.3. Psychosocial Factors

Organizational participation. The physical and psychological benefits of participation in community organizations are significant and have been linked to better health across the lifespan [43,44] as well as reduced mortality risk [45,46]. There are several possible mechanisms through which organizational participation influences health, including facilitating access to resources via diverse network ties [47], promoting healthy behaviors [48], and boosting a sense of identity and purpose in life [49].

Friends. Friendships in adulthood have been associated with a range of positive outcomes, including life satisfaction [50], subjective well-being, and self-rated health [51]. Friends affect health by influencing health behaviors, providing resources and information, as well as by buffering stress [52]. These benefits can have long-term effects. Friendship, including having friends in whom one can confide, has been shown to be longitudinally associated with better mental health [53], lower inflammation [54], greater cognitive functioning [55], and reduced risks of chronic illness [56] and mortality [52].

Depression. Mortality rates are significantly higher among individuals with depression. Much prior research on the effect of depression on mortality focuses on specific clinical cohorts, such as patients with cancer [57] and stroke [58]. However, several studies have reported this association in the general population as well [59,60]. According to one meta-analysis [61], the increased risk of mortality is similar among those with major depressive disorder and subthreshold depression, with both contributing 7% to excess mortality in the population.

### 1.4. Health

Self-rated health. It is well established that individuals’ own health appraisals are highly correlated with objective measures of health among adults of all ages [62,63,64] and highly predictive of mortality [65]. In one study [66], a dose–response pattern between self-rated health and mortality was found to persist over a 30-year period. The predictive power of self-rated health on mortality is not limited to individuals with chronic morbidities [67] but also those without any diagnosed conditions [68]; in many studies, self-rated health remains a significant predictor of mortality even after controlling for health behaviors [69] and indicators of health ascertained with biomarkers [68].

Smoking. Smoking is the leading preventable cause of death in the U.S. [70], with a rate of death 2–3 times higher among current smokers as compared to those who never smoked [71]. Current smokers have a reduced life expectancy by about 10 years [72]. The number of cigarettes smoked per day is associated with mortality [73], though several studies report that smoking duration is a stronger risk factor [74,75].

### 1.5. Financial Factors

Employment status. Paid work has significant health benefits [76,77]. Conversely, unemployment, job loss, and work instability have been shown to predict future physical health problems [78] as well as death [79,80]. In the U.S., many health-promoting resources are tied to employment, including health insurance and retirement benefits [81]. More directly, individuals who are employed have more money which, in turn, provides greater access to both material and non-material resources that are important for health [82].

Net worth. Greater income and wealth are consistent predictors of better health outcomes [83,84]. These associations are observed across the life course [84], though as adults leave the labor market, wealth—or the value of assets minus debt—becomes more reflective of individuals’ financial well-being than income [85]. Among adults in midlife and early old age, having fewer assets and lower levels of wealth is prospectively associated with unhealthier behaviors such as smoking and physical inactivity [85], functional limitations [86], worse self-rated health [87], and higher mortality risk [88,89].

### 1.6. Present Study

Building on this literature, the present study addressed two research questions. First, to what extent is post-secondary education associated with a reduced risk of early mortality (defined as death prior to life expectancy), and does this higher education effect vary by the number of CGG repeats in the *FMR1* gene (a gene x environment interaction)? Second, to what extent are the impacts of post-secondary education and *FMR1* variants on early mortality a function of family, psychosocial, health, and financial advantages that intervene between the completion of education and mortality, or, alternatively, does higher education have a neuroprotective effect? We address these questions using data from the Wisconsin Longitudinal Study (WLS), a unique source of phenotypic data that has been genotyped for CGG repeats in *FMR1*. It is a random sample of a cohort (mostly born in 1939), initially studied as high school seniors in 1957 and subsequently studied periodically [90]. Today, they are over 80 years of age.

We focus on women for the present study for two reasons. First, the majority of *FMR1*-related research has been conducted on males, and there is thus a need to increase knowledge about women who carry CGG expansions. Additionally, women and men differ in longevity in the general population, and therefore separate studies of mortality are needed for women and men.

We hypothesize that post-secondary education will be associated with a reduced risk of early mortality among women in the WLS cohort and that this higher education effect will vary by the number of CGG repeats in the *FMR1* gene. Specifically, we predict that the effect of higher education on mortality will become less pronounced as CGG repeats increase, as a result of the health conditions associated with expanded repeats. In addition, we explore family, psychosocial, health, and financial factors that follow the completion of education and their associations with mortality.

## 2. Materials and Methods

### 2.1. Study Population and Data

All data for the present study were drawn from the WLS, a public-use data set. This study’s population initially consisted of 10,317 women and men who graduated from Wisconsin high schools in 1957. They constituted a one-third random sample of that year’s cohort of high school graduates [90]. Notably, in 1957, approximately 75% of 18-year-olds in Wisconsin graduated from high school. The WLS cohort was followed up in 1975 when they were 36 years old; in 1992 when they were 53 years old; in 2003 when they were 64 years old; and again in 2011 when they were 72 years old. The participants in the 2011 study included 72.2% of the surviving members of the original cohort. Reflecting Wisconsin’s population in the middle of the 20th century, the WLS sample is racially and ethnically homogeneous (99.2% White, 84.2% of Northern European heritage).

Starting in 2007, saliva samples were collected from participants using Oragene kits (DNA Genotek, Inc., Bethlehem, PA, USA). All participants provided informed consent under a protocol approved by the Institutional Review Board of the University of Wisconsin–Madison; per the IRB, the return of genetic results was prohibited. When saliva sample collection was completed, more than two-thirds (69.0%) of surviving WLS members provided samples, which were used for DNA assays including the number of *FMR1* CGG repeats. Those who provided saliva samples had one-half year more schooling (13.8 years vs. 13.3 years, *p* < 0.001) and three points higher IQ scores (102.2 vs. 98.4, *p* < 0.001) than those who did not return saliva samples. Otherwise, they were representative of the WLS sample as a whole.

The analytic sample for the present study began with the 2863 WLS women who provided saliva samples and for whom *FMR1* CGG repeats were assayed. Of these women, 12 had missing data on educational attainment and were excluded from the analytic sample. The remaining 2851 women had CGGs ranging from 7 to 128 repeats. The next highest CGG repeat number among women in this cohort was 84. We decided to exclude the woman with 128 CGGs because including her would have inflated the standard deviation of the CGG repeat variable, leading to less precise estimates from the Cox regression models. Therefore, it was decided to focus the present analysis on participants with 84 or fewer CGG repeats. Reflecting these inclusion criteria, the present analysis was based on 2850 women.

### 2.2. Determination of the FMR1 CGG Triplet Repeat Number

DNA was isolated using standard methods. For saliva samples collected in 2007, following the 2006 wave of the WLS, the number of *FMR1* CGG repeats was determined (under the supervision of author M.W.B.) using a PCR-based protocol that incorporated reagents developed and manufactured by Celera Corporation (Alameda, CA, USA) (see Seltzer et al. [91] for details). Additional saliva samples were collected at the subsequent wave of the WLS. For those samples, the repeat number was determined via an assay using the Asuragen AmplideX^®^ Kit, Austin, TX, USA [92,93], conducted at the Rush University Medical Center Molecular Diagnostics Laboratory (supervised by author E.B.-K.). The results of a concordance study conducted between the two assays (*n* = 22; some from the premutation range and some with normal alleles) indicated that the correlation between the two was 0.9996.

The assays yielded CGG repeat data on the *FMR1* gene on both X chromosomes. Because the WLS genetic data did not include the activation ratio, one X chromosome was selected for analysis in the present study, following the approach we have used previously in population studies of the *FMR1* CGG distribution [94,95]. We selected the longer allele in women who had one expanded (i.e., >40 CGGs) and one normal allele (*n* = 194) and in the four cases who had two expanded alleles. Similarly, we selected the shorter allele in women who had one low allele (i.e., <26 CGGs) and one normal allele (*n* = 872) and in women who had two low alleles (*n* = 138). We randomly selected one allele in women who had two normal alleles (between 26 and 40 CGG repeats, *n* = 1584), and also for those with one low allele and one expanded allele (*n* = 58). Given the preponderance of alleles in the normal range in the randomly selected population studied here, this approach made it possible to probe the effects of the widest range of repeats in the WLS data, ranging from the low end of the distribution (7 CGG repeats) to the higher end of our analytic sample (84 CGG repeats).

CGG repeats were analyzed continuously. However, for descriptive purposes, the sample sizes of repeats in the following categories are as follows: 25 or fewer CGGs (*n* = 1037), 26–40 CGGs (*n* = 1584), and 41–84 CGGs (*n* = 229). The latter category represents the “expansion” range including those in the gray zone (*n* = 215) and premutation (*n* = 14) ranges.

### 2.3. Measures

Table 1 presents the descriptive statistics of the study variables, and Table 2 presents the intercorrelations among the variables.

The key independent variable in this research was the interaction between years of post-secondary education and the number of *FMR1* CGG repeats. Years of post-secondary education ranged from 0 to 9 and was analyzed continuously. Descriptively, the majority of the women in the analytic sample (61%) had no education after high school, while 15.1% had between 1 and 3 years of college, and 23.9% had at least four years of post-secondary education. The dependent variable was mortality status as of 2019, when participants averaged 80 years of age. Variables that might have accounted for the prior likelihood of post-secondary education (IQ score and family SES, both measured during high school) were included in a baseline model. In this cohort, both of these variables were significantly associated with years of post-secondary education (r = 0.415, *p* < 0.001; r = 0.421, *p* < 0.001, respectively), justifying their inclusion as prior controls. Additionally, variables that intervened between the completion of education and mortality status as of 2019 were included in the analytic approach.

Dependent variable. Mortality status in 2019 was the dependent variable. Life expectancy for White women born in 1939 was 67.3 years at birth, but for those who survived until WLS saliva collection (age 68), life expectancy was 85.5 years, as reported by life tables from the Centers for Disease Control and Prevention [96]. Thus, all of the deaths in the present cohort were early deaths (defined as deaths before the cohort’s life expectancy). WLS collected mortality information from the National Death Index and the Social Security Death Index. WLS matched its participants’ records with these sources to obtain mortality status and date of death. The present analysis includes deaths that occurred between 2007 (when saliva was first collected for DNA assays) and December 2019, as deaths prior to saliva collection could not be assayed for CGG repeats. Thus, the survival analysis spanned a 12-year period. Most participants remained alive as of 2019, with 15.5% deceased. The average age at death of the decedents was 75.3.

Baseline controls. Family SES was measured with a weighted composite score of four items: the father’s and mother’s years of education, the father’s occupation using the Duncan Socioeconomic Index [97], and average parental income (see [98] for details). IQ scores were obtained using the Henmon–Nelson Test of Mental Abilities, which was administered during the junior year in high school. IQ scores in the present analysis averaged 102.3 (range = 61 to 145; SD = 14.3).

Intervening Variables. Based on past research (e.g., [42,52,68,89]), four domains were conceptualized as factors intervening between the completion of education and mortality status in 2019: family factors, psychosocial factors, health, and financial factors. The specific variables within each domain that were included in the analyses were selected based on conceptual and empirical criteria. Conceptually, as reviewed above, all the variables were found in past research to be associated with mortality in the general population. Empirically, all the intervening variables were significantly (*p* < 0.05) associated with mortality status in the WLS data when included in a simple hazard model that included only that intervening variable, in addition to years of post-secondary education and CGG repeat number. These variables were measured during three waves of the WLS: in 1975 when the participants were in their 30s (averaging 36 years of age), in 1992 when the participants were in midlife (averaging 53 years of age), and in 2003 when the participants were approaching old age (averaging 64 years of age). These intervening factors include a mix of positive (e.g., close friendships) and negative (e.g., years of smoking) influences on longevity.

Family Factors. Measures from the family domain included being married when participants averaged 36 years of age and whether the participant had experienced the death of their parents before the participant reached midlife. Marital status at age 36 was coded as currently married (1) or any other marital status (0). Parental death prior to midlife was coded as neither parent deceased before 1992 (0), one parent deceased by 1992 (1), or both parents deceased by 1992 (2). We included the measure of parental death to control both shared longevity across the generations and the psychosocial consequences of losing parents before midlife. Note that although 14.1% of participants had a child with a developmental disability or mental health condition, parenting status was not significantly related to maternal mortality and therefore was not included here.

Psychosocial Factors. Measures in this domain included participation in social and civic organizations at age 36, having at least one friend as a confidant in midlife (age 53), and level of depressive symptoms (also measured in midlife). Organizational participation was measured by the number of organizations in which the respondent was a member (from a list of 17 organizations). Friend confidants reflected having at least one friend “with whom you can really share your very private feelings and concerns” (coded 1) or no friend confidant (coded 0). Depressive symptoms in midlife were measured with [99] the Center for Epidemiologic Studies—Depression (CES-D) Scale. Respondents reported the number of days in the past week (0–7 days) on which they experienced each of 20 depression symptoms. The reports were converted to a 4-point scale where 0 = never, 1 = 1 to 2 days, 2 = 3 to 4 days, and 3 = 5 to 7 days, so that the summed total scores matched the conventional scoring of the CES-D. A total score of 16 or higher indicates an elevated risk for clinical depression [99].

Health. The health domain included cumulative years of smoking and self-rated health, both measured when participants were in midlife (age 53). Years of smoking was an indicator of health behaviors. Participants rated their own health as very poor, poor, fair, good, or excellent. We recoded these responses into three categories of self-rated health: unfavorable (coded 0; combining the very poor, poor, and fair categories), good (coded 1), and excellent (coded 2).

Financial Factors. Two financial indicators were included: employment status at age 53, coded as 1 (employed) or 0 (not employed), and net worth at age 64. Net worth was calculated based on two overall categories: (a) the total USD value of equity in home, business, farm, and vehicles as well as retirement accounts, monetary assets (bank accounts, bonds, stocks, mutual funds, etc.), and life insurance value, and (b) the USD value of debts (mortgages and loans). Net worth was defined as the difference between categories a and b, and then dichotomized as the bottom 20% of the sample (1), which was equivalent to having total net worth valued at less than USD 100,000 (in 2003 USD), versus those in the top 80% (0).

### 2.4. Data Analysis

We used Cox proportional hazard models to estimate the effects of the interaction between years of post-secondary education and CGG repeats. We constructed the hazard models in six sequential steps. The baseline model (Model 1) included CGG repeat length and years of post-secondary education, with controls for IQ and family SES. In Model 2, which addressed our first research question, the interaction between CGG repeats and years of post-secondary education was added to Model 1. Subsequently, in Models 3 through 6, variables from each domain of the intervening factors were added in sequence (i.e., family, psychosocial, health, and financial factors). Model 6 addressed our second research question.

Based on prior research, we identified two or three intervening variables per domain that were predictive of early mortality; all of these variables were significant independent predictors of mortality in this cohort. Second, the predictors within each domain were included in a domain-specific Cox regression model, and we retained only those variables for the full analysis that remained significant predictors of mortality, net of the other variables in that domain. We implemented this two-step approach based on prior research employing survival analysis [100,101].

CGG repeat number was centered at 30 repeats (the population mode). IQ was centered at 100 (the population mean). Years of post-secondary education was not centered, as in this cohort 0 years signifies high school completion. The family SES variable and all continuous intervening predictors were centered at their respective means. Parental death and self-rated health were entered into Cox regressions as categorical variables. The proportional hazard assumption for the Cox regression models was tested, and there was no evidence of violation of this assumption. All analyses were conducted using Stata version 17.0 (StataCorp LLC, College Station, TX, USA). The level of significance was set at equal to or less than 0.05.

In post hoc analyses, we utilized three different approaches to visually display the interactive effects of the number of CGG repeats and years of post-secondary education on mortality status. First, for illustrative purposes, we estimated how the relative hazard of mortality changes by each additional year of higher education at three different CGG repeat points: at 25, 30, and 41 repeats. Second, we subsequently estimated the survival functions for women with various levels of post-secondary education (0 years, 2 years, and 4+ years) at 25, 30, and 41 CGG repeats, again for illustrative purposes. Third, we present Kaplan–Meier survival functions between CGG repeats, education, and mortality using the original (raw) data, not adjusting for any covariates. For these post hoc analyses, we selected 25 CGGs, as this number of CGGs has been identified in several studies as the upper limit of the “low zone” [102,103,104,105,106]. We selected 30 CGGs as it is the mode of the population in many published studies. We selected 41 CGGs because, as noted above, it has been identified in many studies as the lower limit of the gray zone.

## 3. Results

### 3.1. Preliminary Domain-Specific Analyses

Table 3 shows the association between the individual variables in each domain and mortality (see column A in Table 3). As described below, six of these variables remained significant when estimated simultaneously with the other variables in their respective domain (see column B in Table 3).

For the family factors domain, being married at age 36 and experiencing the death of both parents before age 53 were significant predictors of mortality when estimated simultaneously (see Table 3, column B). For the psychosocial factors domain, when the three variables in the domain were estimated simultaneously, having at least one friend confidant at age 53 was a significant predictor, but neither organizational participation nor depressive symptoms remained significant predictors of mortality. For the health domain, self-rated health and years of smoking, both measured at age 53, were significant predictors of mortality when estimated simultaneously. From the financial factors domain, net worth at age 64 survived as the significant predictor, but employment status at midlife was not a significant predictor when estimated simultaneously with the net worth variable.

**Figure 1:**
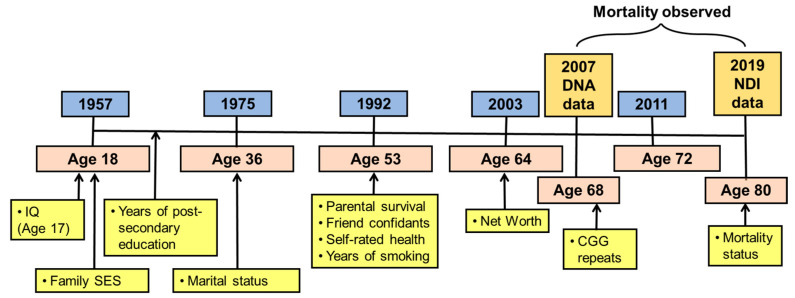
WLS timeline (in blue boxes), average age at timepoints (in pink boxes), and measures (in yellow boxes).

Figure 1 illustrates the timepoint when each variable was measured during the 60+ year course of the WLS as well as the average age of WLS participants at each point of measurement.

### 3.2. Cox Proportional Hazard Models Predicting Mortality

Based on the domain-specific analyses reported in Table 3, we built a six-step multivariate model to determine if the effects of CGG repeats and years of post-secondary education remained significant predictors of early mortality even after the intervening variables were sequentially introduced (see Table 4).

Model 1 in Table 4 portrays the baseline variables (IQ, family SES, CGG repeats, and the main effect of years of post-secondary education). The results indicate that each additional year of post-secondary education significantly reduced the hazard of early mortality by 7%, net of the other variables included in Model 1.

**Figure 2:**
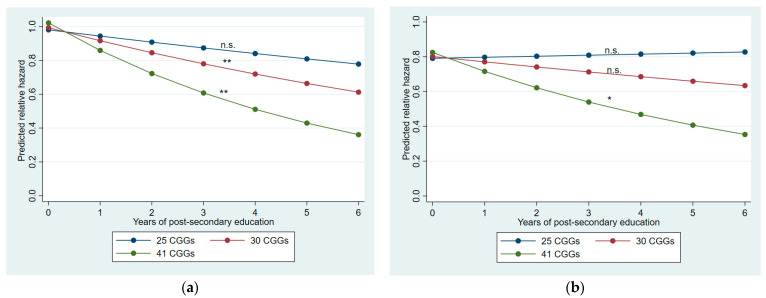
Estimated hazard ratios by education and CGG repeats: (**a**) from Model 2 (with IQ and 1957 family SES controlled); (**b**) from Model 6 (with all intervening factors controlled). Note: n.s. not significant, * *p* < 0.05, ** *p* < 0.01.

Model 2 addressed our first research question, namely whether the effect of post-secondary education on early mortality varied by the number of CGG repeats in *FMR1*. This interaction effect was statistically significant, net of the baseline variables. Figure 2 panel (a) illustrates this interaction effect, showing the hazard of early mortality estimated for women with 25, 30, and 41 repeats. As shown in the figure, the beneficial effects of more years of post-secondary education depended on CGG repeat numbers. At 25 CGG repeats, there was no association between years of post-secondary education and the hazard of early mortality; at 30 repeats, for each additional year of post-secondary education, the hazard of early mortality was reduced by 8%; at 41 repeats, each additional year of post-secondary education reduced the hazard of early mortality by 16% (*p*s < 0.01).

Models 3 through 6 in Table 4 addressed our second research question by sequentially adding the intervening variables. Model 3 added variables from the family factors domain. Being married at age 36 reduced the hazard of early mortality by 28%. By contrast, losing both parents before midlife (as compared to women whose parents were still alive) increased the hazard of early mortality by 54%.

Model 4 added a variable from the psychosocial factors domain, and showed that women who had at least one friend in whom they could confide, as measured at age 53, had a 36% lower hazard of early mortality compared to those with no friend confidant. Note that in Model 4, the effect of marital status (reflecting marital status almost two decades earlier) was no longer a significant predictor of mortality. The effect of parental death, however, remained a significant predictor of early mortality.

Model 5 added two measures from the health domain, namely years of cigarette smoking and self-rated health, both measured in midlife. Each additional year of smoking increased the hazard of mortality by 2%. On average, the duration of cigarette smoking among those who reported having this habit was 21 years, which translates into a 52% increase in the hazard of early mortality. Participants who rated their own health as unfavorable (very poor, poor, or fair health) at age 53 had about 72% higher hazard of early mortality than those who self-rated as having good health. By contrast, those who rated their health as excellent at that point in the life course had about a 32% lower hazard of early mortality than those self-rated as having good health.

The final model (Model 6) brought in the net worth variable measured when participants were aged 64 as an indicator of the financial domain. Women in the bottom 20% of the sample with regard to net worth (or, less than USD 100,000 in total assets as measured in 2003 USD) had a 52% higher hazard of early mortality, net of all other intervening variables in the model.

Notably, the CGG–years of higher education interaction effect remained significant (*p* < 0.05) throughout, providing evidence addressing Research Question 2. This result is illustrated in Figure 2b (above). Here the effect on early mortality was less pronounced than when only baseline variables were included. After including the intervening variables, postsecondary education was not associated with mortality among those with 25 or 30 CGG repeats. However, when estimated at 41 CGG repeats, the effect of higher education on mortality remained significant; for each additional year of post-secondary education, the hazard of early mortality among those with CGG expansions was decreased by 14%, even after all intervening variables were controlled.

**Figure 3:**
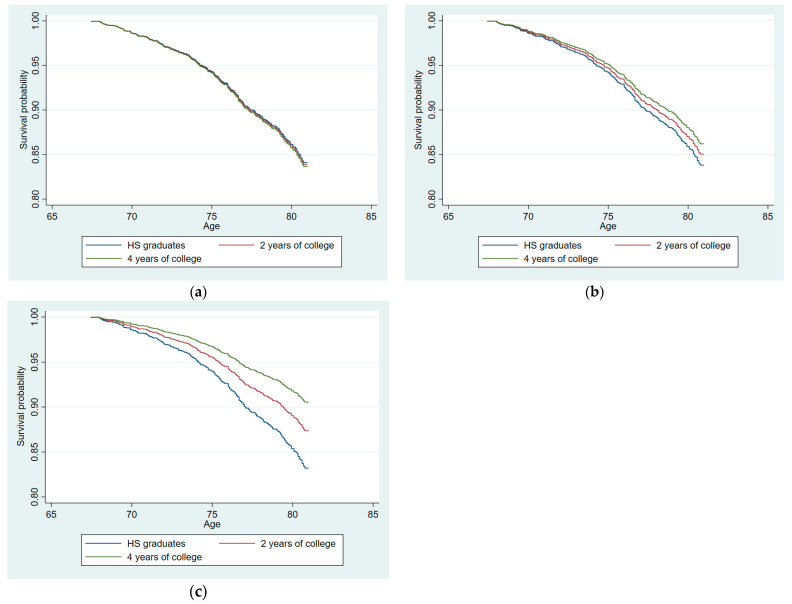
Estimated survival functions by education and CGG repeats (with all intervening factors controlled): (**a**) estimated at 25 CGG repeats; (**b**) estimated at 30 CGG repeats; (**c**) estimated at 41 CGG repeats.

As a further visual display of the survival function (Figure 3), we estimated the function for women with various levels of post-secondary education (0 years, 2 years, and 4+ years). Panels (a) to (c) illustrate the survival functions estimated at 25 repeats, 30 repeats, and 41 repeats, respectively, controlling for all intervening variables. Women with 0 years (i.e., high school graduates) are illustrated with blue lines; women with two years of higher education are illustrated with red lines; women with four or more years of higher education are illustrated with green lines.

As seen in Figure 3a, the three lines were almost completely overlapping, suggesting that for women with 25 CGG repeats, years of higher education had no association with mortality. Approximately 84% of those with 25 repeats were predicted to be alive in 2019, regardless of their level of higher education. In Figure 3b, the survival functions were estimated at 30 CGG repeats, and here the functions diverged by the levels of higher education. For women with 30 CGG repeats and no higher education, about 84% were expected to be alive in 2019. By contrast, about 86% of women with 30 repeats and 4 years of post-secondary education were predicted to be alive in 2019. Figure 3c shows the survival functions estimated at 41 CGG repeats. About 83% of the women with 41 CGG repeats and no post-secondary education were predicted to be alive in 2019, whereas fully 91% of those with 41 CGG repeats and 4 years of post-secondary education were expected to be alive in 2019.

**Figure 4:**
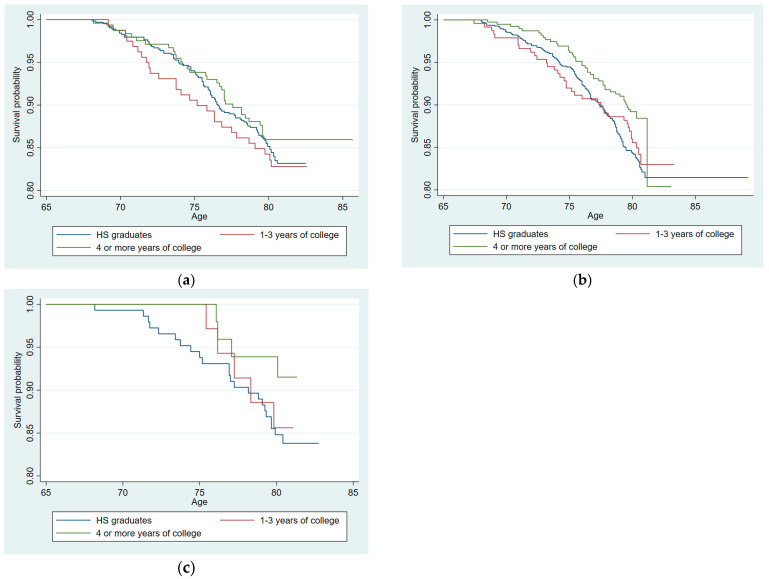
Kaplan–Meier survival functions by education and CGG repeats: (**a**) women with CGG repeats of 25 or lower; (**b**) women with CGG repeats between 26 and 40: (**c**) Women with CGG repeats of 41 or higher.

Figure 4 presents versions of Figure 3 showing the relationships between CGG repeats, education, and mortality using the original (raw) data, not adjusting for any covariates. The Kaplan–Meier survival functions of three subgroups—25 or fewer CGGs, 26–40 CGGs, and 41–84 CGGs—are similar to the adjusted functions shown in Figure 3. In Figure 4, the survival of women with expanded numbers of CGG repeats (Figure 4c) is substantially greater than those with normal-range repeats (26–40 CGGs), as well as those with 25 or fewer repeats.

## 4. Discussion

Consistent with much past research conducted on the general population, in the present study the main effect of higher education was found to be associated with a lower risk of mortality, before the intervening factors were introduced. This positive effect of higher education has been observed in a number of studies of *FMR1* premutation carriers, where higher education (as a main effect) has been associated with reduced risk of health and cognitive symptoms [30,31,32,33,34,107,108].

The present study extended past research on *FMR1* CGG repeats with a number of methodological contributions: the individuals analyzed here were a random sample of a specific cohort rather than a clinically-identified sample; the study evaluated a wide range of *FMR1* CGG repeats, ranging from 7 to 84 repeats; it focused on women, whereas much of the previous *FMR1* research focused on men; it controlled for prior factors that might have accounted for the likelihood of higher education (IQ score and family SES); and it examined whether the higher education effect was moderated by *FMR1* CGG repeat number—a gene X environment interaction effect.

We hypothesized that there would be a significant interaction between CGG repeat number and years of higher education in the prediction of early mortality, and this hypothesis was supported by the data. However, the direction of the interaction effect was not what we had expected. We predicted that women who had greater numbers of CGG repeats would benefit less from higher education than those with repeats in the normal range. This hypothesis was based on the health risks associated with the *FMR1* CGG expansions, including FXTAS, FXPOI, FXAND, and other specific health conditions. This hypothesis was also influenced by a previous study of gray zone males in the general population where elevated rates of death after life expectancy were observed [13]. Notably, that study focused on the mortality of men after their life expectancy, whereas our study assessed the mortality of women prior to their life expectancy. Nevertheless, this was a relevant prior study, and it affected our hypothesis.

However, we found the opposite of our prediction: the benefit of higher education was most pronounced in women who had a greater number of CGG repeats. Illustrating the point, controlling for IQ and family SES measured in high school, the effect of each additional year of post-secondary education was twice as large in those with 41 or more CGG repeats than in those with 30 repeats. Notably, most of the women in the present study who had 41 or more CGG repeats were in the gray zone of *FMR1*. Substantially less is known about the genotype–phenotype associations of those in the gray zone than in the premutation range (for exceptions, see [12,109,110,111]), which is another contribution of the present research.

Importantly, although we presented the original data without controlling for any possibly confounding factors (see Figure 4), the essential goal of the present study was to adjust the original data for possible factors that would confound or nullify the effect of higher education on mortality. As shown in Table 4 and Figure 3, we adjusted for IQ score and family SES as measured during high school to control for the differential access to higher education. We adjusted for subsequent other factors reflecting cumulative life course advantages following the completion of education (family, psychosocial, health, and financial factors). Controlling for multiple indicators of such advantages, the interaction effect between higher education and CGG repeats remained significant over six decades of the adult life course. Importantly, although these intervening variables were associated with early mortality, the effect of the CGG–education interaction in predicting early mortality nevertheless remained significant even with these factors controlled.

Why might this be the case? We interpret this in the context of differential sensitivity to the environment, a conceptualization that posits that some people are more reactive or sensitive than the norm to both negative and positive environmental conditions. This conceptualization aims to explain why people react differently to the same environment. In the context of *FMR1* variants, past research has investigated how negative aspects of the environment (e.g., stressful life events, stressful parenting) differentially affect women with expanded CGG repeats, depending on their number of repeats within the premutation range [112,113].

However, very few past studies have examined whether positive aspects of the environment also have this differential sensitivity effect. One exception [108] found evidence suggestive of differential sensitivity to positive emotional support among mid-size premutation carriers as compared to those with higher or lower numbers of repeats within the premutation range. Another indication of differential sensitivity in response to positive aspects of the environment [114] focused on women with premutation CGG repeats; this study reported that women (mean age of 45) who had between 70 and 100 repeats and who had a college degree had better cognitive functioning than those with fewer or a greater number of CGG repeats within the premutation range.

The benefits of higher education have been robustly demonstrated in research on the general population as well as in clinical samples, with the benefits interpreted as evidence of neuroprotection [27]. The intellectual stimulation of higher education is said to build cognitive reserve during late adolescence and early adulthood, a time of neuroplasticity. Cognitive reserve increases resilience to subsequent hardships and ultimately can reduce the risk and severity of neurodegeneration and other health vulnerabilities. The present research extends past studies by demonstrating that the neuroprotective effect of higher education is stronger among highly educated women with greater numbers of *FMR1* CGG repeats.

The present study controlled for selection effects that might have affected the likelihood of going to college (IQ and family SES during high school) as well as multiple variables that occurred subsequently during the decades of adulthood. Nevertheless, the neuroprotective effect of higher education among those with expanded CGG repeats persisted, providing evidence of the lasting benefit of higher education, particularly among women with expanded repeats. The sample was population-based rather than clinically identified. Since only a small proportion had a child with a developmental disability, the study was able to largely disaggregate stressful parenting effects from genetic susceptibility, thus offering a clearer view of the genetic effect than possible in clinical studies. Furthermore, none of the women in the present study were aware of their own genetic status and thus this source of possible bias was eliminated.

The present study can offer clinical implications for women with expanded CGG repeats, both for those in the gray zone and those in the low premutation range. Higher education is an investment that has lifelong benefits that may possibly reduce the risks that such women may experience. Their daughters who might also have gray zone or premutation repeats might be encouraged to pursue higher education or other forms of cognitive enrichment during late adolescence and early adulthood for the same reason.

However, the present study was not without limitations. It was based on one cohort from a single state, and nearly all participants were White and of Northern European heritage. Future research replicating these results in different populations is needed. Much has changed since the women in this study finished high school in 1957. In 1960, 38% of women who completed high school enrolled in college, whereas in 2020 the percentage was 66% [115]. Thus, replications of this research with subsequent birth cohorts will further clarify the association between higher education and mortality in the context of greater access to educational opportunities, and the effect of CGG repeats on this association.

Furthermore, this is a study of women, and future research is needed to learn how higher education may benefit men with varying numbers of *FMR1* CGG variants. All deaths that were studied here were early deaths, as they preceded the life expectancy of the cohort; future research on this cohort that extends after age 85 will reveal whether the longevity advantage enjoyed by women with CGG expansions and who achieved higher education continues past the average life expectancy of the cohort. An additional limitation is that deaths that occurred prior to the time when DNA samples were obtained could not be included in the present analysis, and thus the conclusions are limited to deaths that occurred between the ages of 68 and 80. Finally, this study lacked data on activation ratios, and therefore future research with access to activation ratios is needed to replicate this work when the active allele can be identified.

## 5. Conclusions

The present study suggested that the neuroprotective effect of higher education enjoyed by women with 41 or more CGG repeats (at least up to 84) persisted across 60 years, even when factors such as IQ, family SES, intergenerational family ties, having close friends, good health, and sufficient financial resources are controlled. These women with higher repeats were unexpectedly more likely to have avoided early mortality. Genetic variants such as *FMR1* CGG expansions are often evaluated in terms of the risks they pose, but the present results extend past research suggesting that such risks can be reduced or offset when the environment is enriched.

## Figures and Tables

**Table 1:** Descriptive statistics of study variables.

	(*n*)	Mean (s.d.) [Min, Max]/Percentages (*n*)
Mortality (1 = deceased)	2850	15.5% (441)
Age at death	439	75.3 (3.4) [67, 81]
Years of post-secondary education	2850	1.4 (2.1) [0, 9]
CGG repeats	2850	29.2 (6.8) [7, 84]
IQ (age 17)	2850	102.3 (14.3) [61, 145]
Family SES (age 18)	2850	16.3 (11.2) [1, 97]
**Family Factors**		
Marital status (age 36) (1 = married)	2743	89.8% (2464)
Parental deaths (age 53)	2783	0. no parental death: 18.0% (502) 1. one parent deceased: 46.2% (1286) 2. both parents deceased: 35.8% (995)
**Psychosocial Factors**		
Organizational participation (age 36)	2740	2.3 (1.7) [0, 10]
Having friend confidants (age 53) (1 = yes)	2431	85.6% (2080)
Depressive symptoms (CES-D) (age 53)	2422	8.9 (7.9) [0, 54]
**Health**		
Self-rated health (age 53)	2452	0. unfavorable ^a^: 9.4% (231) 1. good: 58.9% (1444) 2. excellent: 31.7% (777)
Years of smoking (age 53)	2410	9.9 (13.1) [0, 50]
Years of smoking among smokers (age 53)	1132	21.1 (11.4) [<1, 50]
**Financial Factors**		
Employed (age 53) (1 = yes)	2783	80.1% (2230)
Net worth (age 64) (1 = bottom 20% of the sample)	2679	19.8% (531)

Note: ^a^. The “unfavorable” health category includes three categories of self-rated health: “very poor,” “poor,” and “fair”.

**Table 2:** Correlations among the study variables.

	IQ	Family SES	CGG Repeats	Years of Post-Secondary Education	Married	Parental Deaths	Friend Confidants	Smoking	Self-Rated Health	Net Worth	Mortality Status
IQ	1.000										
Family SES	0.309 ***	1.000									
CGGs	−0.037 *	0.020	1.000								
Education	0.415 ***	0.421 ***	−0.005	1.000							
Married	−0.057 **	−0.029	−0.001	−0.138 ***	1.000						
Parent deaths ^a^	−0.041 *	−0.087 ***	−0.020	−0.073 ***	0.021	1.000					
Friend confidants	0.003	0.066 **	0.040	0.047 *	0.003	−0.004	1.000				
Smoking	−0.020	0.023	−0.035	−0.080 ***	−0.026	0.036	0.026	1.000			
Health ^a^	0.077 ***	0.124 ***	0.011	0.148 ***	0.018	−0.013	0.060 *	−0.062 **	1.000		
Net worth	−0.144 ***	−0.093 ***	−0.021	−0.100 ***	−0.102 ***	0.004	−0.020	0.046 *	−0.109 ***	1.000	
Mortality	−0.038 *	−0.016	−0.013	−0.054 **	−0.034	0.052 *	−0.067 ***	0.140 ***	−0.114 ***	0.098 ***	1.000

Note: * *p* < 0.05, ** *p* < 0.01, *** *p* < 0.001. ^a^ The correlation coefficients were calculated using the original ordinal coding (0, 1, 2), although these variables were treated as categorical variables in Cox regressions.

**Table 3:** Intervening variables’ effects on mortality by life course domains ^a^.

	Column A: Main Effect of Each Variable When Estimated Separately ^b^	Column B: Main Effect of Each Variable When Estimated Simultaneously ^c^
1. Family Factors		
Marital status (age 36)	0.73 * [0.54, 0.97]	0.72 * [0.53, 0.97]
Parental deaths (age 53)		
(ref. group: no parental death)		
one parent deceased	1.30 + [0.97, 1.73]	**1.32 [0.98, 1.77]**
both parents deceased	1.46 * [1.09, 1.96]	1.54 ** [1.13, 2.08]
2. Psychosocial Factors		
Organizational participation (age 36)	0.93 * [0.88, 0.99]	0.95 [0.89, 1.01]
Friend confidants (age 53)	0.66 ** [0.51, 0.86]	0.67 ** [0.51, 0.88]
Depressive symptoms (age 53)	1.01 * [1.00, 1.03]	1.01 [0.99, 1.02]
3. Health		
Years of smoking (age 53)	1.03 *** [1.02, 1.03]	1.02 *** [1.02, 1.03]
Self-rated health (age 53)		
(ref. group: good health)		
unfavorable (very poor/poor/fair)	1.64 ** [1.23, 2.19]	1.66 ** [1.24, 2.22]
excellent	0.65 ** [0.50, 0.84]	0.67 ** [0.51, 0.87]
4. Financial Factors		
Employed (age 53)	0.80 * [0.64, 1.00]	0.81 [0.64, 1.02]
Net worth (age 64)	1.68 *** [1.35, 2.08]	1.70 *** [1.36, 2.11]

Note: + *p* < 0.10, * *p* < 0.05, ** *p* < 0.01, *** *p* < 0.001. ^a^ Hazard ratios are presented with 95% confidence intervals in brackets. ^b^ The Cox regressions for each individual intervening variable in column A also included CGG repeats and years of post-secondary education. ^c^ For the domain-specific models in column B, all intervening variables from the domain were included simultaneously along with the interaction between CGG repeats and years of post-secondary education. Variables that remained significant in the domain-specific models are bolded.

**Table 4:** Interaction effects of education and CGG repeats on mortality with selected intervening life course variables ^a^.

	Model 1: Baseline	Model 2: + [CGG × Edu.]	Model 3: + [Family Factors]	Model 4: + [Psychosocial Factors]	Model 5: + [Health]	Model 6: + [Financial Factors]
IQ (age 17)	1.00 [0.99, 1.00]	1.00 [0.99, 1.00]	1.00 [0.99, 1.01]	1.00 [0.99, 1.01]	1.00 [0.99, 1.01]	1.00 [0.99, 1.01]
Family SES (age 18)	1.00 [0.99, 1.01]	1.00 [0.99, 1.01]	1.01 [0.99, 1.02]	1.00 [0.99, 1.02]	1.00 [0.99, 1.01]	1.00 [0.99, 1.01]
CGG repeats	1.00 [0.99, 1.01]	1.00 [0.99, 1.02]	1.00 [0.99, 1.02]	1.00 [0.98, 1.02]	1.00 [0.98, 1.02]	1.00 [0.98, 1.02]
Years of post-secondary education	0.93 * [0.88, 0.99]	0.92 *** [0.87, 0.98]	0.92 ** [0.86, 0.97]	0.92 * [0.86, 0.98]	0.94 [0.88, 1.01]	0.96 [0.90, 1.03]
CGG × Yrs. PS Edu	--	0.99 * [0.98, 1.00]	0.99 * [0.98, 1.00]	0.99 * [0.98, 1.00]	0.99 * [0.98, 1.00]	0.99 * [0.98, 1.00]
Married (age 36)	--	--	0.72 * [0.53, 0.97]	0.81 [0.58, 1.13]	0.82 [0.59, 1.16]	0.87 [0.61, 1.22]
Parental deaths (age 53) (ref.: no parental death)						
one parent deceased	--	--	1.31 [0.97, 1.77]	1.20 [0.87, 1.65]	1.24 [0.86, 1.66]	1.24 [0.88, 1.73]
both parents deceased	--	--	1.54 ** [1.14, 2.10]	1.42 * [1.03, 1.97]	1.43 * [1.03, 2.00]	1.43 * [1.02, 2.01]
Friend confidants (age 53)	--	--	--	0.64 ** [0.49, 0.84]	0.64 ** [0.48, 0.84]	0.67 ** [0.50, 0.89]
Years of smoking (age 53)	--	--	--	--	1.02 *** [1.01, 1.03]	1.02 *** [1.01, 1.03]
Self-rated health (age 53) (ref.: good health)						
unfavorable	--	--	--	--	1.72 *** [1.27, 2.32]	1.70 *** [1.24, 2.30]
excellent	--	--	--	--	0.68 *** [0.52, 0.89]	0.69 *** [0.52, 0.91]
Net worth (age 64)	--	--	--	--	--	1.52 ** [1.18, 1.97]
(*n*)	2850	2850	2676	2348	2305	2212

Note: * *p* < 0.05, ** *p* < 0.01., *** *p* < 0.001. ^a^ Hazard ratios are presented with 95% confidence intervals in brackets.

## Data Availability

The datasets analyzed for this study can be found at https://researchers.wls.wisc.edu/data/survey-data/.
